# Multimodal Deep Learning Integrating Tumor Radiomics and Mediastinal Adiposity Improves Survival Prediction in Non‐Small Cell Lung Cancer: A Prognostic Modeling Study

**DOI:** 10.1002/cam4.71077

**Published:** 2025-08-04

**Authors:** Ye Niu, Han‐bing Xie, Hao‐bo Jia, Lin Zhao, Le Liu, Ping‐ping Liu, Xue‐meng Li, Rui‐tao Wang, Yuan‐zhou Li

**Affiliations:** ^1^ Department of Internal Medicine Harbin Medical University Cancer Hospital, Harbin Medical University Harbin Heilongjiang China; ^2^ The School of Computer Science and Engineering University of Electronic Science and Technology of China Chengdu Sichuan China; ^3^ Department of Radiology Harbin Medical University Cancer Hospital, Harbin Medical University Harbin Heilongjiang China

**Keywords:** computerized tomography, deep learning, mediastinal fat, non‐small cell lung cancer, survival

## Abstract

**Background and Purpose:**

Prognostic stratification in non‐small cell lung cancer (NSCLC) presents considerable challenges due to tumor heterogeneity. Emerging evidence has proposed that adipose tissue may play a prognostic role in oncological outcomes. This study investigates the integration of deep learning (DL)–derived computed tomography (CT) imaging biomarkers with mediastinal adiposity metrics to develop a multimodal prognostic model for postoperative survival prediction in NSCLC patients.

**Methods:**

A retrospective cohort of 702 surgically resected NSCLC patients was analyzed. Tumor radiomic features were extracted using a DenseNet121 convolutional neural network architecture, while mediastinal fat area (MFA) was quantified through semiautomated segmentation using ImageJ software. A multimodal survival prediction model was developed through feature‐level fusion of DL‐extracted tumor characteristics and MFA measurements. Model performance was evaluated using Harrell's concordance index (C‐index) and receiver operating characteristic (ROC) analysis. Risk stratification was performed using an optimal threshold derived from training data, with subsequent Kaplan–Meier survival curve comparison between high‐ and low‐risk cohorts.

**Results:**

The DL‐based tumor model achieved C‐indices of 0.787 (95% CI: 0.742–0.832) for disease‐free survival (DFS) and 0.810 (95% CI: 0.768–0.852) for overall survival (OS) in internal validation. Integration of MFA with DL‐derived tumor features yielded a multimodal model demonstrating enhanced predictive performance, with C‐indices of 0.823 (OS) and 0.803 (DFS). Kaplan–Meier analysis revealed significant survival divergence between risk‐stratified groups (log‐rank *p* < 0.05).

**Conclusion:**

The multimodal fusion of DL‐extracted tumor radiomics and mediastinal adiposity metrics represents a significant advancement in postoperative survival prediction for NSCLC patients, demonstrating superior prognostic capability compared to unimodal approaches.

## 1 Introduction

According to 2020 global cancer statistics, lung cancer accounted for 2.2 million new cases, ranking second in incidence and first in mortality worldwide [1]. Surgical resection remains the cornerstone of curative treatment for non‐small cell lung cancer (NSCLC), yet postoperative survival outcomes exhibit marked heterogeneity due to multifactorial influences [2, 3]. Accurate preoperative survival prediction is therefore critical for optimizing therapeutic decision‐making and personalized care.

Body composition metrics, particularly adipose tissue distribution, have emerged as prognostic determinants in oncology [4, 5]. While the L3 cross‐sectional area on abdominal computed tomography (CT) is considered the gold standard for body composition assessment [6, 7], its clinical utility in NSCLC is limited by the predominant use of chest CT protocols that often exclude L3 [8]. Mediastinal fat, a metabolically active chest cavity component, has recently been implicated as a prognostic biomarker in NSCLC through its association with systemic inflammation and tumor progression [9, 10, 11, 12].

Deep learning (DL) offers transformative potential in oncology by autonomously extracting high‐dimensional radiomic features from medical images, circumventing limitations of manual feature selection in traditional radiomics [13]. Prior studies have validated DL's utility in predicting lymph node metastasis and treatment response in NSCLC [14, 15], yet its integration with adipose tissue metrics for survival prediction remains unexplored.

This study pioneers the development of a multimodal prognostic model combining DL‐derived tumor radiomics with mediastinal fat area (MFA) quantification to predict survival outcomes in resected NSCLC patients.

## 2 Materials and Methods

The institutional review board gave its approval to this study (approval number: YD2024‐06). Because the study was designed retrospectively, the requirement for informed consent was waived.

### 2.1 Study Design and Participants

**FIGURE 1:**
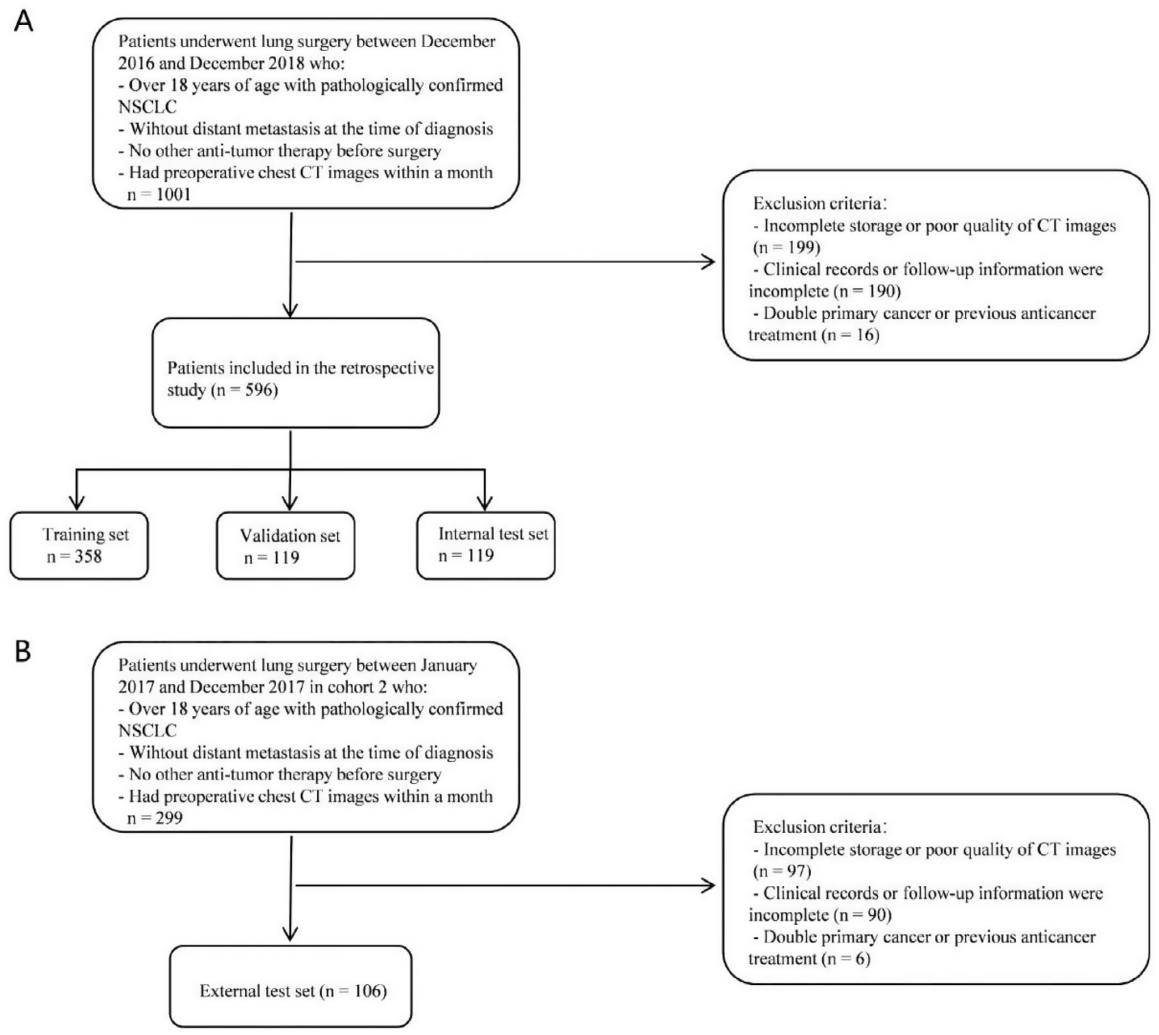
Flow diagram of patient inclusion and exclusion criteria. (A) cohort 1; (B) cohort 2.

**FIGURE 2:**
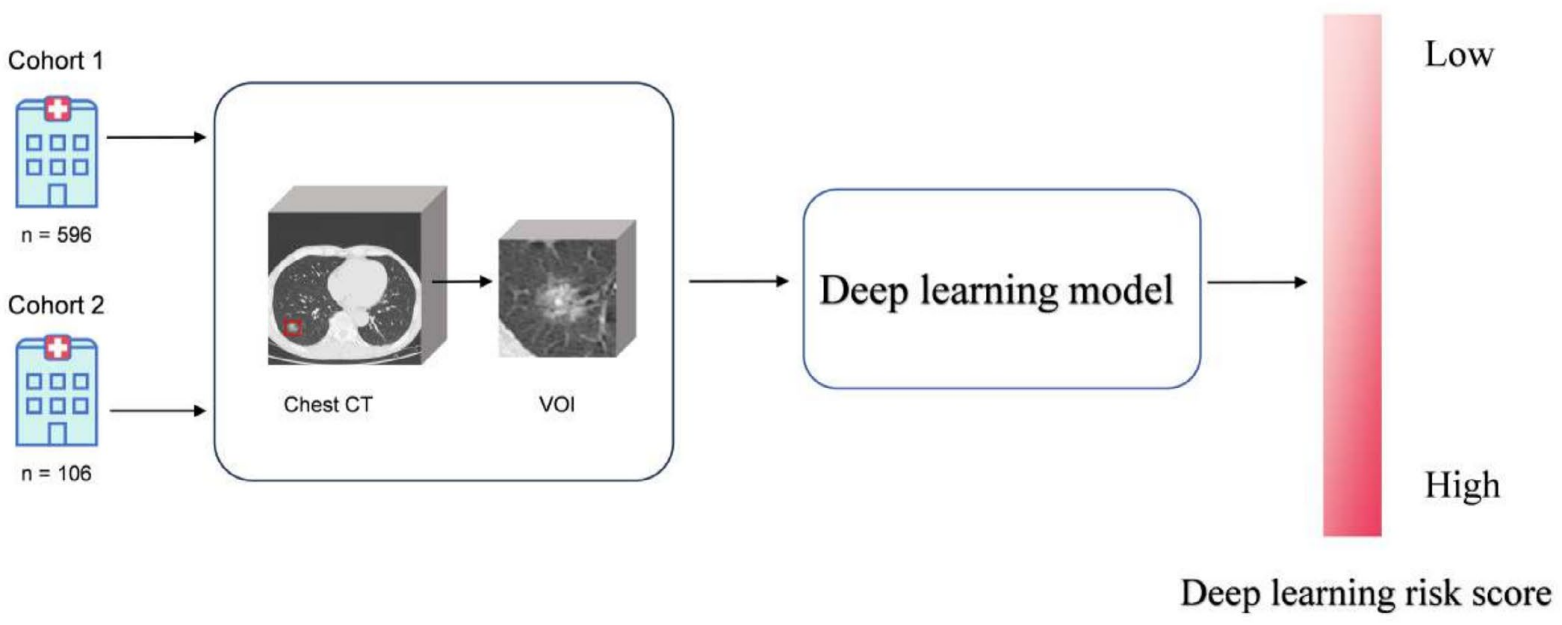
Workflow for the deep learning model.

This retrospective multicenter study analyzed 702 NSCLC patients undergoing surgical resection between December 2016 and December 2018. Cohort 1 (*n* = 596) was randomly divided into training (*n* = 358), validation (*n* = 119), and internal test (*n* = 119) sets. Cohort 2 (*n* = 106; January to December 2017) served as an independent external test set. Figure 1 depicts the patient recruiting procedure as well as the inclusion/exclusion criteria. Figure 2 depicts the workflow for the DL model.

### 2.2 Clinical Characteristics

Demographic, pathological, and serum biomarker data were extracted from electronic records. Primary endpoints were 5‐year overall survival (OS) and disease‐free survival (DFS), calculated from surgery to death/recurrence.

### 2.3 Image Acquisition and Preprocessing

**FIGURE 3:**
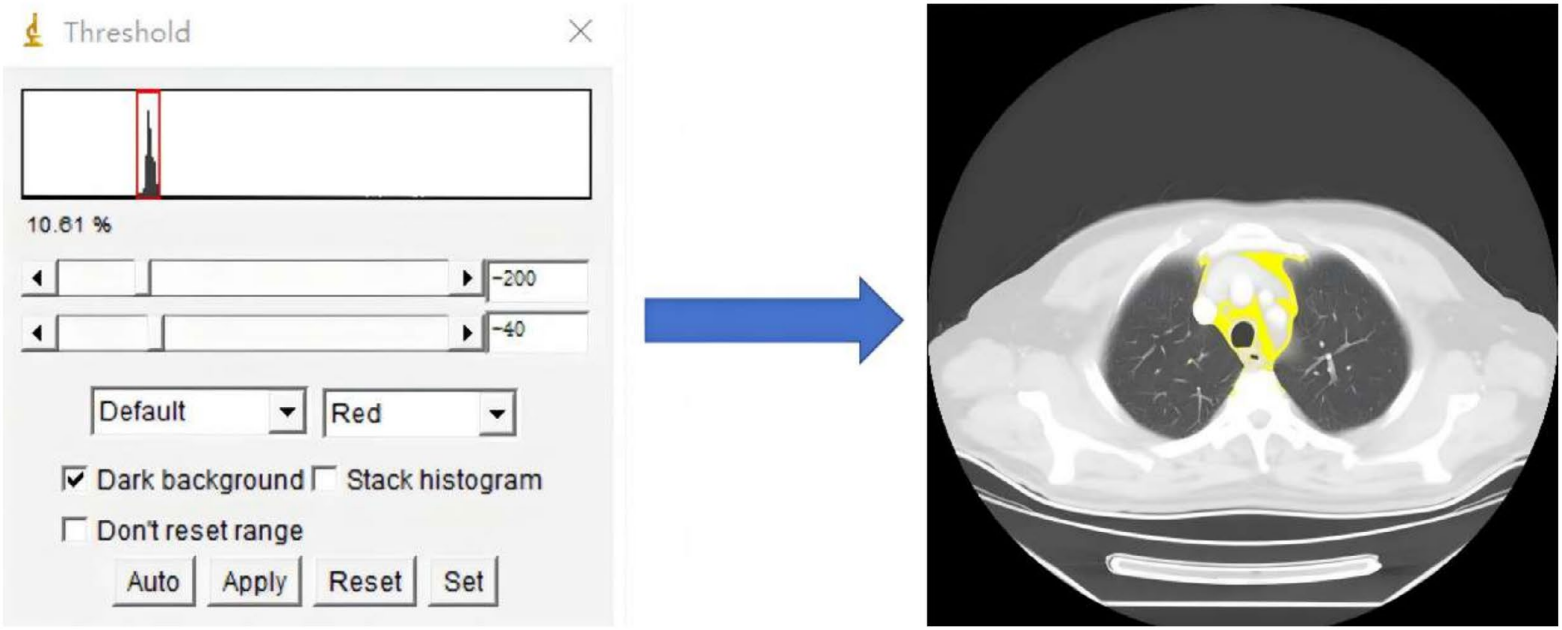
Application example of mediastinal fat boundary contouring. The yellow line represents the mediastinal fat area.

Each patient had an enhanced CT scan performed within 2 weeks prior to the surgery. The parameters are listed in Appendix method 1 and Table S1. MFA was quantified as a two‐dimensional measurement at the first layer upward of the aortic arch, with anatomical boundaries defined as follows: anteriorly by the sternum, posteriorly by vertebral bodies, and laterally by mediastinal pleura and great vessels [16]. Tissue segmentation was based on preestablished thresholds of HU in the range of −200 to −40 [17]. MFA measurements in the chest CT sections were manually drawn by two radiologists to use Image J software (Figure 3) (Appendix method 2).

### 2.4 Deep Learning Architecture

Tumor volumes of interest (VOIs; 150^3^ voxels) underwent preprocessing: resampling (0.6 × 0.6 × 0.6 mm^3^), rotation (±180°), translation (±3 pixels), sharpening/blurring augmentation, and intensity normalization. DenseNet‐121 was selected as the optimal architecture after comparative analysis, generating a deep learning risk score (DLRS) via Cox‐optimized loss functions for OS/DFS prediction (Appendix method 3) [18, 19]. In brief, the parameters of the model were iteratively updated in the direction that lowered the loss by calculating the gradient using the two losses for OS and DFS that were computed and added at a 1:1 ratio [20]. We used an attention map for heatmap synthesis and gradient‐weighted class activation mapping to visualize the regions of the image that were crucial for prediction.

### 2.5 Multimodal Model Construction

**FIGURE 4:**
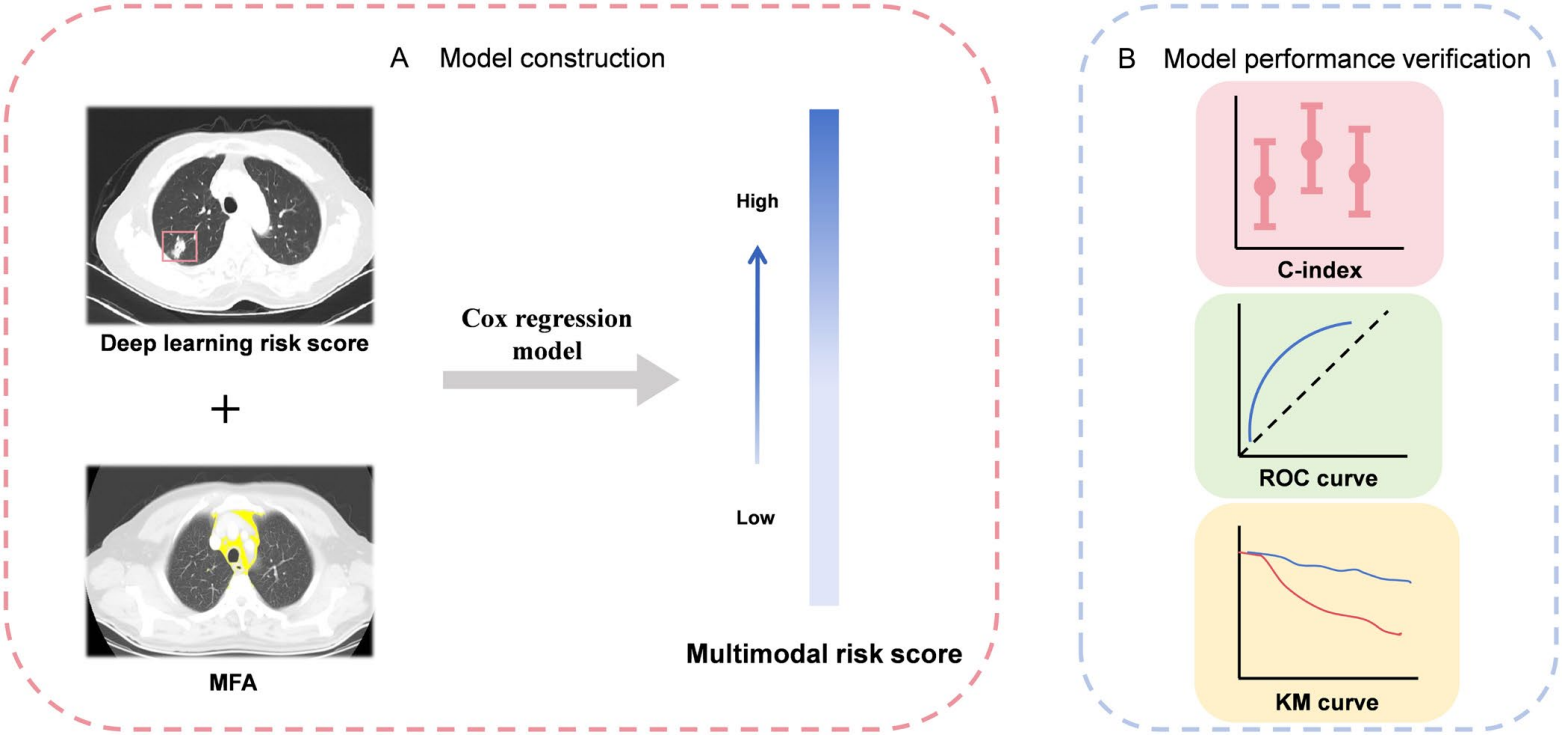
Workflow for the multimodal model. (A) Model construction; (B) Model performance verification.

Cox regression integrated DLRS with MFA and clinical variables to produce a multimodal risk score (MRS) (Appendix method 4), as shown in Figure 4. Risk stratification thresholds maximized Kaplan–Meier survival differences in the training set.

### 2.6 Statistical Analysis

The chi‐square test and fisher exact test were used to assess categorical variables, while the student *t*‐test and Mann–Whitney *U* test were performed to compare continuous variables. Variables with *p* < 0.05 in univariate Cox regression analysis were then subjected to multivariate Cox regression analysis to identify the independent prognostic factors for OS and DFS. The Harrell C‐index and the area under the receiver operating characteristic curve (AUC) were used to evaluate the prognostic performance of the model. The Kaplan–Meier method was applied to generate survival curves for survival, and the log‐rank test was used to compare between groups. The cutoff values were established to compute the performance metrics and define the risk groups based on the optimal cutoff value that maximized the difference in the Kaplan–Meier survival curve in the training set. All analyses were performed in IBM SPSS (version 26), Python (version 3.9.13), and R software (version 4.1.0). *p* < 0.05 was deemed statistically significant in all two‐sided statistical tests. All R code and data are publicly available (https://github.com/Strawberry666666/NSCLC‐prediction‐code).

## 3 Results

### 3.1 Patient Characteristics

The sample size of the training set was 358, with 119 in the validation set, and the internal and external test sets consisted of 119 and 106, respectively. The mean age was 57.2 years; 392 cases (55.8%) were female, 578 cases (82.3%) were adenocarcinoma, 124 cases (17.7%) were squamous cell carcinoma, and others. Table 1 displays the baseline features of data sets. Except for the pathological type, the distribution of the other baseline characteristics did not vary significantly. Patients were further split into low‐ and high‐risk groups; Tables S2 and S3 display the clinical features of these two groups. For OS, MFA, T stage, and N stage were statistically significant. For DFS, there were significant differences in T stage, N stage, NSE, and MFA.

**TABLE 1:** Baseline analysis of different sets of NSCLC patients.

Variables	Training set	Validation set	*p*	Internal test set	*p*	External test set	*p*
Number	358	119		119		106	
OS (months)			0.165		0.756		0.622
Mean ± SD	56.5 ± 9.8	54.7 ± 12.7		56.2 ± 10.8		57 3 ± 9.3	
DFS (months)			0.380		0.144		0.810
Mean ± SD	51.2 ± 18.0	49.5 ± 19.8		48.1 ± 20.3		50.7 ± 18.1	
Age in years			0.879		0.107		0.759
Mean ± SD	57.4 ± 8.4	57.6 ± 7.8		55.8 ± 9.9		57.1 ± 8.9	
BMI (kg/m^2^)			0.340		0.025		0.272
Mean ± SD	24.0 ± 3.1	23.7 ± 3.0		23.3 ± 3.4		24.4 ± 3.3	
NSE (ng/mL)			0.400		0.095		0.133
Media (Q1–Q3)	13.9 (12.1–15.9)	13.5 (11.9–15.1)		14.3 (12.3–16.2)		14.4 (12.4–16.5)	
CEA (ng/mL)			0.075		0.926		0.672
Media (Q1–Q3)	2.4 (1.5–4.2)	2.0 (1.2–3.6)		2.6 (1.6–4.0)		2.5 (1.3–4.0)	
MFA (cm^2^)			0.319		0.148		0.198
Mean ± SD	5.7 ± 2.9	6.0 ± 3.1		5.2 ± 2.5		6.1 ± 3.1	
Sex			0.725		0.849		0.051
Female	204 (57.0)	70 (58.8)		69 (58.0)		49 (46.2)	
Male	154 (43.0)	49 (41.2)		50 (42.0)		57 (53.8)	
Smoking history			0.538		0.770		0.077
Yes	139 (38.8)	50 (42.0)		48 (40.3)		52 (49.1)	
No	219 (61.2)	69 (58.0)		71 (59.7)		54 (50.9)	
T stage			0.798		0.085		0.196
T1	285 (79.6)	93 (78.2)		86 (72.3)		76 (71.7)	
T2	54 (15.1)	20 (16.8)		23 (19.3)		23 (21.7)	
T3	16 (4.5)	4 (3.4)		5 (4.2)		7 (6.6)	
T4	3 (0.8)	2 (1.6)		5 (4.2)		0 (0.0)	
N stage			0.331		0.062		0.171
N0	283 (79.1)	91 (76.5)		82 (68.9)		76 (71.7)	
N1	35 (9.8)	9 (7.6)		15 (12.6)		11 (10.4)	
N2	40 (11.1)	19 (15.9)		22 (18.5)		19 (17.9)	
Pathological type			0.001		0.108		0.001
AD	313 (87.4)	89 (74.8)		97 (81.5)		79 (74.5)	
Others	45 (12.6)	30 (25.2)		22 (18.5)		27 (25.5)	
EGFR			0.755		0.070		0.944
Positive	98 (27.4)	35 (29.4)		22 (18.5)		28 (26.4)	
Negative	260 (72.6)	84 (70.6)		97 (81.5)		78 (73.6)	
Chemotherapy			0.255		0.062		0.689
Yes	79 (22.1)	33 (27.7)		37 (31.1)		26 (24.5)	
No	279 (77.9)	86 (72.3)		82 (68.9)		80 (75.5)	

*Note:* *p* values indicate the comparisons of the difference between the training set and the validation, internal and external test sets, respectively.

Abbreviations: AD, adenocarcinoma; BMI, body mass index; CEA, carcinoma embryonic antigen; DFS, disease‐free survival; EGFR, epidermal growth factor receptor; MFA, mediastinal fat area; NSCLC, non‐small cell lung cancer; NSE, neuron‐specific enolase; OS, overall survival.

### 3.2 Basic DL Model Prediction: Results, Validation, and Visualization

The basic DL model for extracting CT image features of the target tumor lesion contributes significantly to the final predictive classification. In order to select which model performed the best, we compared the predictive performance of DenseNet121, ResNet‐50, ResNet‐101, DenseNet‐169, VGG‐16, and Inception‐v3 (Table 2). In the test set, the C‐index of the DenseNet‐121 model for predicting OS and DFS was 0.810 and 0.787, respectively, which was higher than other 3D CNN models. Therefore, DenseNet‐121 was chosen as the encoder for CT images of tumors. Using the internal test set, the AUC of the DLRS, that output from the DenseNet‐121 model, was 0.884 (95% CI: 0.789–0.979) and 0.819 (95% CI: 0.693–0.945), respectively, indicating the accuracy of the prediction for 3‐ and 5‐year OS. In the internal test set, the DLRS was similarly successful in properly predicting DFS at 3 years (0.806 [0.696, 0.916]) and 5 years (0.814 [0.715, 0.913]). Additionally, we tested the model on an independent external test cohort (Table S4).

**TABLE 2:** C‐index comparison of different deep learning models.

Models	OS			DFS		
	T	V	I‐T	T	V	I‐T
ResNet‐50	0.773 (0.740–0.805)	0.753 (0.697–0.809)	0.722 (0.668–0.777)	0.814 (0.791–0.837)	0.795 (0.758–0.831)	0.762 (0.722–0.801)
ResNet‐101	0.744 (0.709–0.779)	0.742 (0.685–0.799)	0.691 (0.638–0.743)	0.801 (0.776–0.826)	0.784 (0.726–0.842)	0.757 (0.703–0.811)
DenseNet‐121	0.937 (0.926–0.948)	0.865 (0.838–0.893)	0.810 (0.757–0.864)	0.908 (0.893–0.923)	0.885 (0.862–0.907)	0.787 (0.749–0.825)
DenseNet‐169	0.944 (0.936–0.951)	0.838 (0.796–0.881)	0.790 (0.741–0.839)	0.934 (0.921–0.947)	0.831 (0.789–0.873)	0.785 (0.743–0.827)
VGG‐16	0.738 (0.702–0.773)	0.670 (0.608–0.732)	0.667 (0.615–0.720)	0.745 (0.708–0.782)	0.732 (0.691–0.773)	0.690 (0.635–0.745)
Inception‐v3	0.697 (0.659–0.735)	0.664 (0.600–0.729)	0.655 (0.597–0.713)	0.707 (0.622–0.792)	0.686 (0.612–0.760)	0.665 (0.610–0.720)

*Note:* 95% confidence intervals included in brackets.

Abbreviations: DFS, disease‐free survival; I‐T, independent test set; OS, overall survival; T, training set; V, validation set.

**FIGURE 5:**
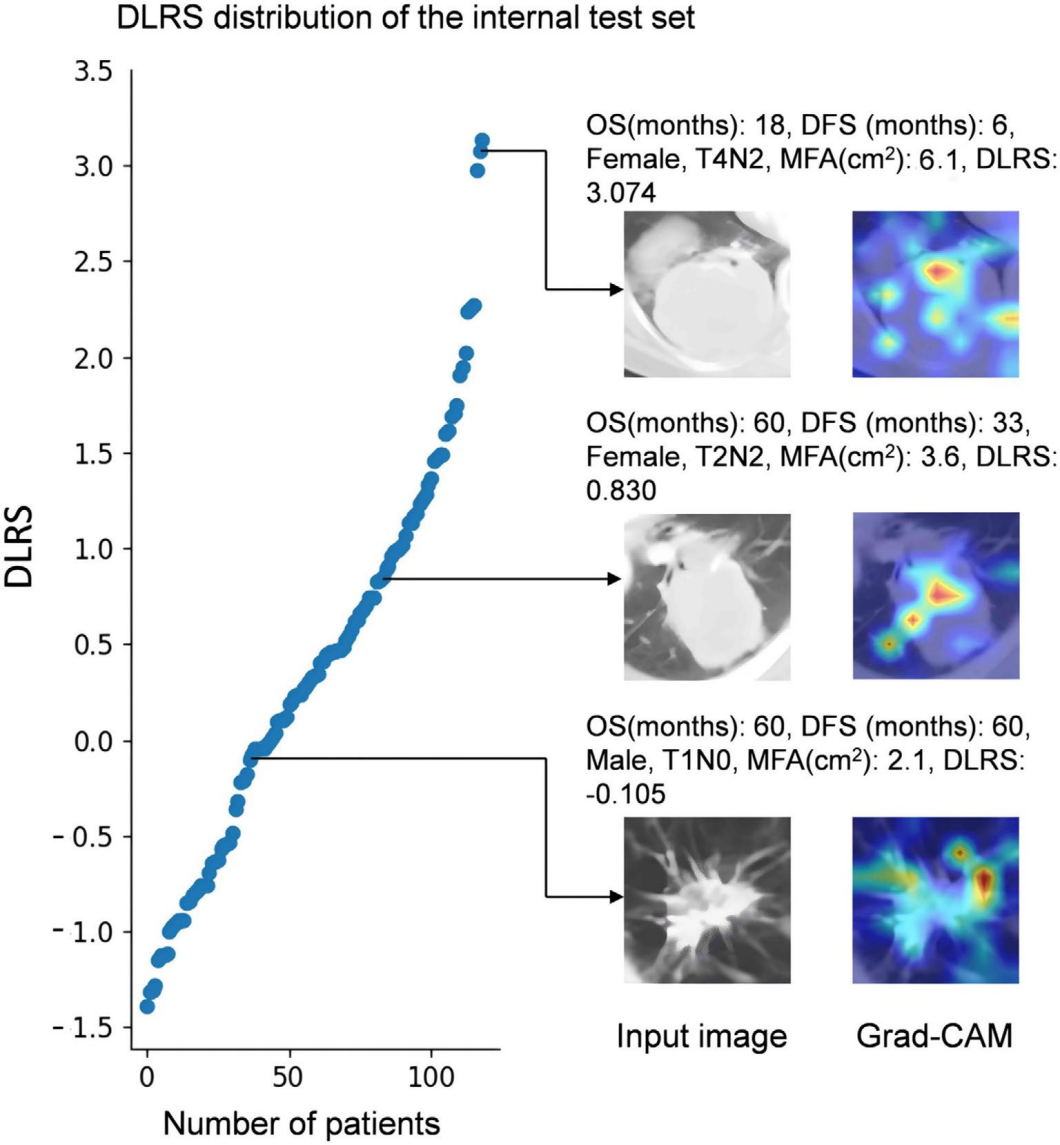
Scatter plot showing the distribution of deep learning risk score (DLRS) and illustrations of three representative patients.

In addition, the DenseNet model has a lower number of parameters (Figure S1). The histogram of the DLRS distribution and gradient‐weighted activation maps overlaid with CT images showed that DLRS focused attention on tumor regions, as shown in Figure 5.

### 3.3 Multivariable Cox Regression

As shown in Tables 3 and 4, the multivariate Cox regression analysis for OS displayed that the HR for DLRS was 7.876 (95% CI: 5.421–12.012; *p* < 0.001) in the training set. For DFS, the HR was 6.430 (95% CI: 4.668–8.012; *p* < 0.001). Similarly, for OS, the HR for MFA was 2.417 (95% CI: 1.922–3.446; *p* = 0.001). For DFS, the HR for MFA was 2.027 (95% CI: 1.512–2.626; *p* = 0.001). It can be seen that the HRs of DLRS and MFA were higher than those of other clinical factors; DLRS and MFA were independent factors for predicting survival.

**TABLE 3:** The Cox regression results for overall survival in the training set.

Variables	Univariable analysis		Multivariable analysis	
	HR (95% CI)	*p*	HR (95% CI)	*p*
Sex (female vs. male)	0.668 (0.384, 1.164)	0.155		
Age (years)	1.022 (0.987, 1.058)	0.218		
Smoking history (yes vs. no)	1.505 (0.864, 2.621)	0.149		
BMI (kg/m^2^)	0.948 (0.864, 1.041)	0.262		
T stage (T3 + T4 vs. T1 + T2)	3.445 (1.617, 7.341)	0.001	2.321 (1.335, 3.083)	0.019
N stage (N1 + N2 vs. N0)	5.365 (3.073, 9.363)	< 0.001	1.417 (0.755, 2.240)	0.335
Pathological type (AD vs. others)	0.410 (0.214, 0.785)	0.007	1.052 (0.514, 2.153)	0.889
NSE (ng/mL)	1.053 (0.994, 1.114)	0.079		
CEA (ng/mL)	1.004 (1.002, 1.007)	0.001	0.831 (0.542, 1.036)	0.062
EGFR (positive vs. negative)	1.032 (0.539, 1.974)	0.925		
Chemotherapy (yes vs. no)	0.791 (0.451, 0.968)	0.032	0.673 (0.492, 1.063)	0.100
MFA (cm^2^)	1.129 (1.050, 1.213)	0.001	2.417 (1.922, 3.446)	0.001
DLRS (H vs. L)	6.223 (4.595, 8.427)	< 0.001	7.876 (5.421, 12.012)	< 0.001

Abbreviations: AD, adenocarcinoma; BMI, body mass index; CEA, carcinoembryonic antigen; DLRS, deep learning risk score; EGFR, epidermal growth factor receptor; H, high‐risk; L, low‐risk; MFA, mediastinal fat area; NSE, neuron‐specific enolase.

**TABLE 4:** The Cox regression results for disease‐free survival in the training set.

Variables	Univariable analysis		Multivariable analysis	
	HR (95% CI)	*p*	HR (95% CI)	*p*
Sex (female vs. male)	0.669 (0.424, 1.056)	0.084		
Age (years)	1.002 (0.975, 1.030)	0.887		
Smoking history (yes vs. no)	1.437 (0.910, 2.270)	0.120		
BMI (kg/m^2^)	0.979 (0.907, 1.056)	0.580		
T stage (T3 + T4 vs. T1 + T2)	3.269 (1.677, 6.373)	0.001	1.269 (0.972, 1.533)	0.085
N stage (N1 + N2 vs. N0)	6.251 (3.944, 9.907)	< 0.001	1.470 (0.824, 1.982)	0.158
Pathological type (AD vs. others)	0.542 (0.303, 0.970)	0.039	1.280 (0.884, 2.668)	0.210
NSE (ng/mL)	1.034 (0.981, 1.090)	0.216		
CEA (ng/mL)	1.006 (1.003, 1.008)	< 0.001	1.522 (0.861, 2.713)	0.274
EGFR (positive vs. negative)	1.131 (0.671, 1.905)	0.645		
Chemotherapy (yes vs. no)	0.689 (0.329, 0.952)	0.040	0.798 (0.420, 1.231)	0.124
MFA (cm^2^)	1.105 (1.038, 1.177)	0.002	2.027 (1.512, 2.626)	0.001
DLRS (H vs. L)	6.005 (4.626, 7.795)	< 0.001	6.430 (4.668, 8.012)	< 0.001

Abbreviations: AD, adenocarcinoma; BMI, body mass index; CEA, carcinoembryonic antigen; DLRS, deep learning risk score; EGFR, epidermal growth factor receptor; H, high‐risk; L, low‐risk; MFA, mediastinal fat area; NSE, neuron‐specific enolase.

### 3.4 Model Performance

In the internal test set, the C‐index of the multimodal model for predicting OS was 0.823 (95% CI: 0.773–0.870), which was higher than that of DLRS alone (0.810 [95% CI: 0.757–0.864]) and MFA alone (0.628 [95% CI: 0.564–0.692]). Moreover, the C‐index predicted by the multimodal model was higher than that of DLRS and MFA alone in all other sets (Tables 5 and 6). The AUC of the multimodal model in the internal test set for predicting 3‐year OS and 5‐year OS was 0.864 (95% CI: 0.775–0.953) and 0.840 (95% CI: 0.717–0.963), respectively. Similarly, for DFS, the AUCs were 0.825 (95% CI: 0.724–0.926) and 0.838 (95% CI: 0.742–0.934), respectively (Table S5).

**TABLE 5:** C‐index of different models predicting overall survival.

Models	T	*p*	V	*p*	I‐T	*p*	E‐T	*p*
DL	0.937 (0.926–0.948)	< 0.001	0.865 (0.838–0.893)	0.010	0.810 (0.757–0.864)	0.003	0.759 (0.692–0.825)	0.104
MFA	0.649 (0.613–0.685)	< 0.001	0.680 (0.633–0.727)	< 0.001	0.628 (0.564–0.692)	0.023	0.691 (0.628–0.755)	0.040
Multimodal	0.946 (0.935–0.956)	Ref	0.887 (0.861–0.913)	Ref	0.823 (0.773–0.870)	Ref	0.786 (0.729–0.843)	Ref

*Note:* 95% confidence intervals included in brackets. The “survcomp” package was used for calculating and comparing C‐indexes.

Abbreviations: DL, deep learning; E‐T, external test set; I‐T, independent test set; MFA, mediastinal fat area; T, training set; V, validation set.

**TABLE 6:** C‐index of different models predicting disease‐free survival.

Models	T	*p*	V	*p*	I‐T	*p*	E‐T	*p*
DL	0.908 (0.893–0.923)	0.004	0.885 (0.862–0.907)	0.003	0.787 (0.749–0.825)	0.003	0.701 (0.652–0.750)	0.014
MFA	0.627 (0.596–0.657)	< 0.001	0.633 (0.588–0.678)	< 0.001	0.590 (0.535–0.646)	< 0.001	0.683 (0.635–0.731)	0.219
Multimodal	0.913 (0.898–0.928)	Ref	0.893 (0.872–0.915)	Ref	0.803 (0.766–0.839)	Ref	0.728 (0.682–0.774)	Ref

*Note:* 95% confidence intervals included in brackets. The “survcomp” package was used for calculating and comparing C‐indexes.

Abbreviations: DL, deep learning; E‐T, external test set; I‐T, independent test set; MFA, mediastinal fat area; T, training set; V, validation set.

**FIGURE 6:**
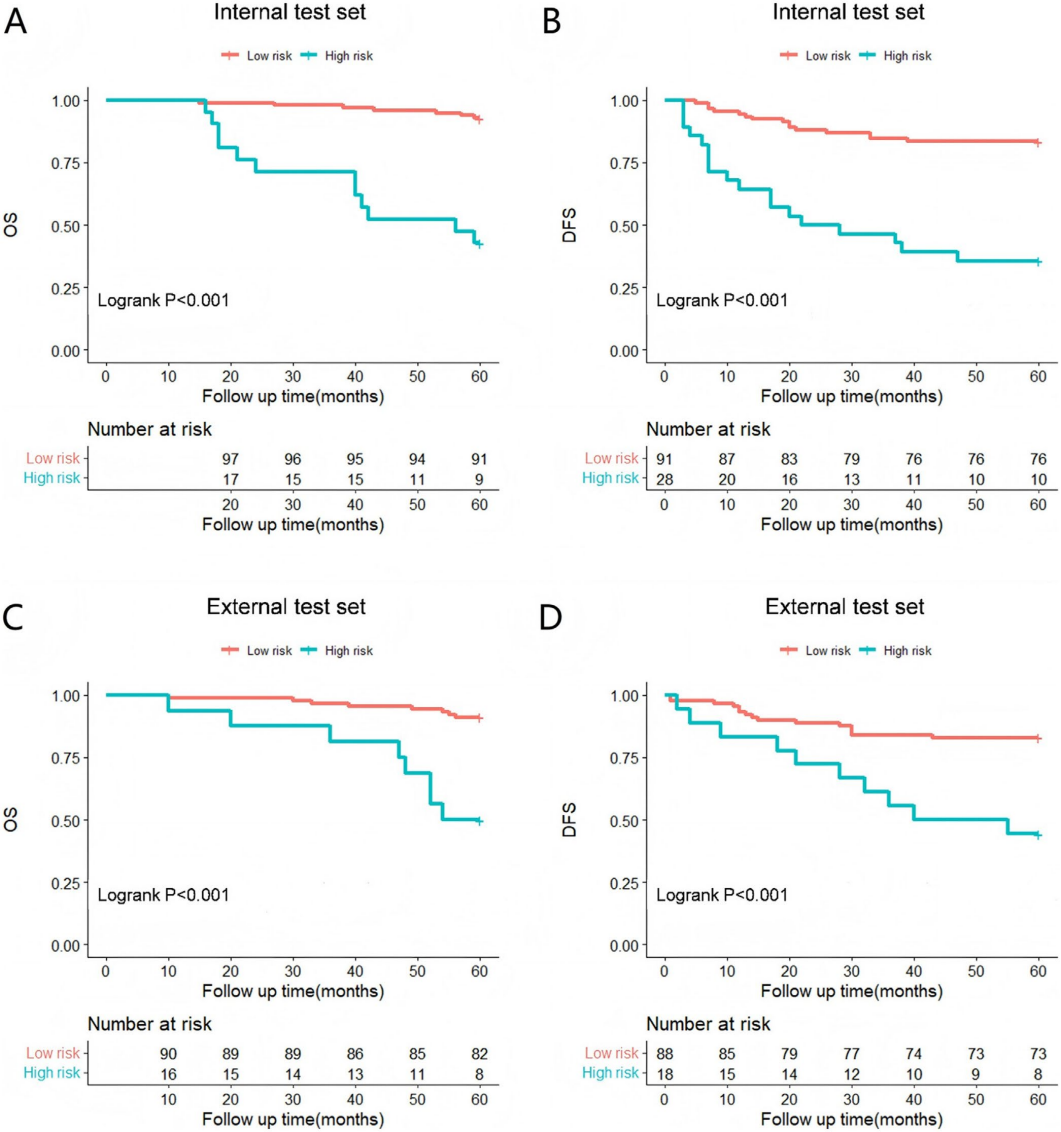
Kaplan–Meier survival analysis. (A) Kaplan‐Meier survival curves for overall survival (OS) in the internal test set. (B) Kaplan‐Meier survival curves for disease‐free survival (DFS) in the internal test set. (C) Kaplan‐Meier survival curves for overall survival (OS) in the external test set. (D) Kaplan‐Meier survival curves for disease‐free survival (DFS) in the external test set.

To refine prognostic risk stratification, patients were divided into low‐risk and high‐risk groups using the MRS. For OS and DFS, the MRS cutoff values were calculated as 5.82 and 9.81, respectively. The two groups' variations in survival are shown in Figure 6 and Figure S2, with worse survival in the high‐risk group.

## 4 Discussion

We developed a 3D multimodal model to predict NSCLC patients' survival. The multimodal model constructed by MFA combined with DLRS can significantly improve the accuracy of NSCLC survival prediction.

This study establishes the first 3D multimodal model integrating DL‐derived tumor radiomics with MFA for NSCLC survival prediction, achieving superior accuracy. MRS could enhance preoperative risk stratification by identifying patients at high risk of occult metastasis or early recurrence. For example, a patient with elevated MRS might benefit from neoadjuvant therapy to target micrometastases prior to resection. Conversely, low‐risk patients could proceed directly to surgery, avoiding delays from unnecessary systemic therapy. Attention maps further refine surgical margins by highlighting peritumoral regions with radiomic signatures of microinvasion. Surgeons could use these maps intraoperatively, paired with real‐time imaging, to ensure complete resection of high‐risk zones. In addition, patients stratified as high‐risk could undergo intensified postoperative surveillance, including 3‐monthly PET‐CT scans and circulating tumor DNA (ctDNA) monitoring, rather than standard 6‐month intervals. This mirrors the trial, where ctDNA detection and follow‐up might reduce recurrence‐related mortality in early‐stage NSCLC [21]. Similarly, elevated MRS (reflecting pro‐inflammatory MFA) might prompt early medical services to mitigate adiposity‐driven comorbidities.

Unlike prior studies, limited by manual feature selection [13] or posttreatment imaging requirements [14], our DL framework leverages pretreatment CT data, enhancing clinical feasibility. The Cox‐optimized loss function addressed censored data challenges, avoiding oversimplification through survival time binarization [22]. More notably, increasing evidence suggests that changes in adipose tissue are associated with disease progression in cancer patients [23, 24]. The research conducted by Yao Lu and colleagues revealed that a lifetime of exposure to excessive amounts of visceral adipose tissue (VAT) can enhance the risk of lung cancer [25]. More VAT was generally linked to a worse chance of survival for patients with colorectal and pancreatic malignancies, according to an analysis [26]. Therefore, on the basis of using DL to extract tumor features for predicting survival in NSCLC patients, we have selected mediastinal adipose tissue as the second biomarker for patients and combined it to construct a multimodal model. The model outperformed the DL model alone. It is suggested that mediastinal adipose tissue contains more information that is helpful to predict the survival of NSCLC.

It's still unclear exactly how adipose tissue influences survival in terms of biological mechanisms. Unlike the characteristics of subcutaneous adipose and visceral adipose in other parts of the body, the human mediastinal adipose tissue depot may have characteristics similar to brown adipose tissue (BAT) that affect its metabolic characteristics [27]. BAT within the mediastinum secretes adipokines such as leptin and pro‐inflammatory cytokines (e.g., IL‐6, TNF‐α), which promote tumor cell proliferation, angiogenesis, and immune evasion [28, 29]. Leptin, overexpressed in hypertrophic adipocytes, activates JAK/STAT and PI3K/AKT pathways in NSCLC cells, driving chemoresistance and metastatic potential [30]. Conversely, BAT‐mediated thermogenesis may exacerbate systemic cachexia by elevating energy expenditure, thereby worsening patient frailty [31]. Furthermore, peritumoral adipocytes engage in bidirectional cross talk with NSCLC cells via extracellular vesicles carrying miRNAs (e.g., miR‐155, miR‐21) that reprogram tumor metabolism and suppress CD8^+^ T‐cell activity [32, 33]. MFA also recruits tumor‐associated macrophages through CCL2 and CCL5 signaling, fostering an immunosuppressive niche conducive to metastasis [34]. These mechanisms collectively position MFA as a dynamic regulator of NSCLC aggressiveness and postoperative recurrence.

We employed Grad‐CAM to visualize regions of CT images that most strongly influenced the DL‐derived tumor radiomics predictions. This spatial mapping helps clinicians understand how anatomical/tumor features (e.g., necrosis, spiculation) contribute to risk stratification. Although our multimodal model achieved good prognostic performance, its clinical adoption will require rigorous validation of explainability tools in prospective settings. Future work may explore the integration of additional interpretability methods, such as SHAP, to further enhance clinical trust and understanding. As underscored by Doshi‐Velez et al. [35], the accountability of AI systems under regulatory frameworks necessitates not only predictive accuracy but also auditability of decision‐making processes. In line with this principle, we have operationalized accountability through open‐sourced code, rigorous algorithmic auditing, and reproducible pipeline documentation to enable external validation of model behavior.

While traditional machine learning (ML) models, such as those employed by Koyama et al. [36], provide transparent prognostic insights through feature weighting or rule‐based outputs, their capacity to model complex, nonlinear interactions in high‐dimensional imaging data remains limited. Our DL methodology prioritizes the extraction of latent tumor radiomic patterns—such as texture heterogeneity and necrosis—that are imperceptible to manual feature engineering. The superior performance of DL comes at the cost of interpretability. Clinicians may favor ML models for treatment personalization (e.g., selecting targeted therapies based on EGFR status), whereas DL's imaging‐driven risk stratification could better guide adjuvant therapy in postoperative settings where occult metastatic risk dominates decision‐making. Future NSCLC prognostication systems could integrate ML's interpretable biomarkers with DL's imaging insights, enabling clinicians to validate data‐driven predictions against known biology.

The study has some limitations. First, while our multimodal model demonstrates prognostic utility, its clinical implementation requires prospective validation of interpretability frameworks to meet regulatory standards for AI‐based decision support. Second, the semiautomatic fat measurement protocol introduced modest inter‐reader variability (mean Dice score = 0.85 ± 0.07), primarily at vascular/bony interfaces requiring manual correction [37, 38]. This reproducibility gap may limit clinical translation compared to fully automated systems. Third, the 2016–2018 study period preceded both (a) clinical adoption of PD‐L1 testing in resected NSCLC and (b) modern adjuvant therapies (targeted agents/immunotherapy), preventing biomarker‐stratified survival analyses. Finally, observed survival outcomes reflect historical chemotherapy‐dominated paradigms rather than current standards (e.g., osimertinib/alectinib in adjuvant settings), as most targeted therapies were administered postprogression.

## 5 Conclusions

The integration of DL‐based tumor radiomics with mediastinal adiposity metrics enables accurate preoperative survival stratification in NSCLC. This multimodal approach advances personalized oncology by informing risk‐adapted treatment strategies.

## Author Contributions

Conceptualization: Ye Niu, Han‐bing Xie, and Rui‐tao Wang. Methodology: Ye Niu and Hao‐bo Jia. Software: Ye Niu and Hao‐bo Jia. Data curation: Han‐bing Xie, Lin Zhao, Le Liu, Ping‐ping Liu, Xue‐meng Li, and Rui‐tao Wang. Investigation: Ye Niu, Lin Zhao, and Ping‐ping Liu. Validation: Ye Niu. Formal analysis: Ye Niu. Supervision: Ye Niu, Han‐bing Xie, Hao‐bo Jia, Lin Zhao, Le Liu, Xue‐meng Li, Rui‐tao Wang, and Yuan‐zhou Li. Funding acquisition: Rui‐tao Wang. Visualization: Ye Niu and Hao‐bo Jia. Project administration: Han‐bing Xie and Rui‐tao Wang. Resources: Rui‐tao Wang and Yuan‐zhou Li. Writing – original draft: Ye Niu and Hao‐bo Jia. Writing – review and editing: Ye Niu, Hao‐bo Jia and Rui‐tao Wang. All authors have read and approved the final version of the manuscript.

## Ethics Statement

This study was performed in line with the principles of the Declaration of Helsinki. Approval was granted by the Ethics Committee of Harbin Medical University Cancer Hospital (approval number: YD2024‐06).

## Conflicts of Interest

The authors declare no conflicts of interest.

## Supporting information


**Figure S1.** Performance parameters of various deep learning models.

**Figure S2.** Kaplan–Meier survival analysis in training and validation sets.

**Table S1.** The CT image acquisition parameters of the two centers. **Table S2.** Characteristics of patients stratified by high and low risk according to multimodal risk score for predicting overall survival. **Table S3.** Characteristics of patients stratified by high and low risk according to multimodal risk score for predicting disease‐free survival. **Table S4.** Deep learning model prediction performance. **Table S5.** Multimodal model prediction performance.

**Appendix S2.** Supplementary Methods for Image Analysis and Modeling.

## Data Availability

Some of the datasets generated and/or analyzed during the current study are not publicly available but are available from the corresponding author upon reasonable request.
