# Supplementary figures and images for: Multimodal Deep Learning Integrating Tumor Radiomics and Mediastinal Adiposity Improves Survival Prediction in Non‐Small Cell Lung Cancer: A Prognostic Modeling Study
Source: Cancer Med. 2025 Aug 4;14(15):e71077. doi: 10.1002/cam4.71077 (PMC12319420; doi:10.1002/cam4.71077)

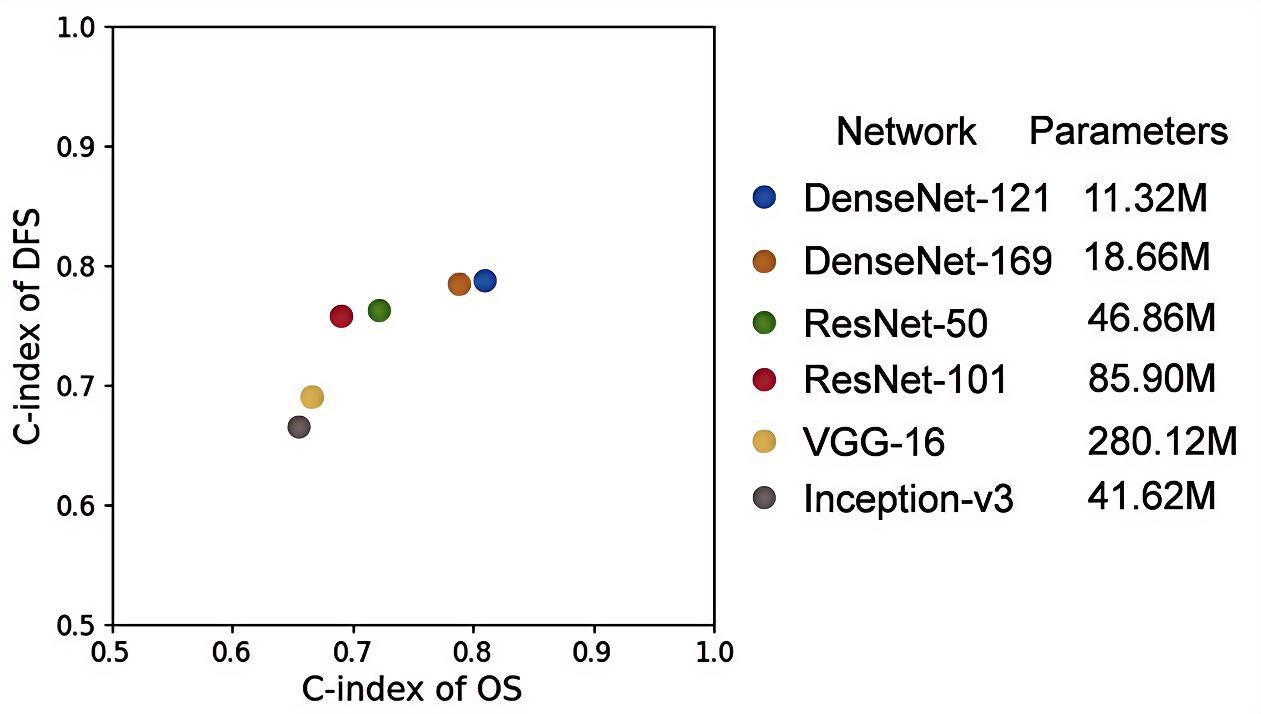

Supplement: Supplementary file 1 — Figure S1. Performance parameters of various deep learning models. [file CAM4-14-e71077-s001.jpg]

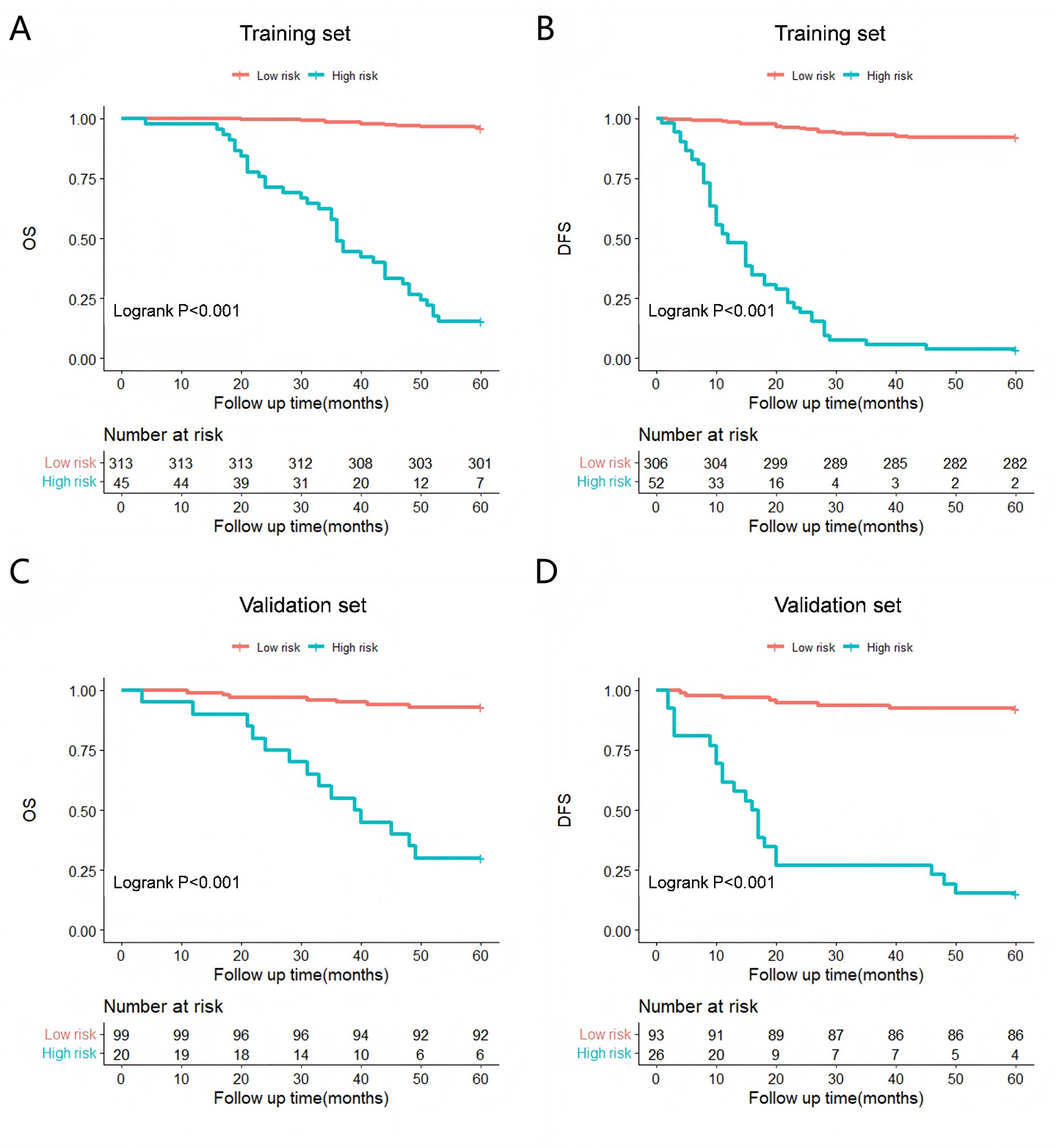

Supplement: Supplementary file 2 — Figure S2. Kaplan–Meier survival analysis in training and validation sets. [file CAM4-14-e71077-s003.jpg]
