# Supplementary material for: Multimodal Deep Learning Integrating Tumor Radiomics and Mediastinal Adiposity Improves Survival Prediction in Non‐Small Cell Lung Cancer: A Prognostic Modeling Study
Source: Cancer Med. 2025 Aug 4;14(15):e71077. doi: 10.1002/cam4.71077 (PMC12319420; doi:10.1002/cam4.71077)
Supplement: Supplementary file 3 — Table S1. The CT image acquisition parameters of the two centers. Table S2. Characteristics of patients stratified by high and low risk according to multimodal risk score for predicting overall survival. Table S3. Characteristics of patients stratified by high and low risk according to multimodal risk score for predicting disease‐free survival. Table S4. Deep learning model prediction performance. Table S5. Multimodal model prediction performance. [file CAM4-14-e71077-s002.docx]

**Table S1**. **The CT image acquisition parameters of the two centers**

|  |  | **Center 1** | **Center 2** |
| --- | --- | --- | --- |
|  | Parameters | Harbin Medical University Cancer Hospital | The Second Affiliated Hospital, Harbin Medical University |
| **CT system**  **information** | CT system | 256-slice spiral CT (SOMATOM Definition Flash, Siemens Healthineers, Germany) or a 64-slice spiral CT (Brilliance 64, PHILIPS, Netherlands) or a 16-slice spiral CT (BrightSpeed, GE Medical Systems, USA) | 256-slice spiral CT (Brilliance iCT, ROYAL PHILIPS, Netherlands ) or a 64-slice spiral CT (Discovery CT750 HD scanner, GE Medical Systems, USA) or a 16-slice spiral CT (SOMATON Sensation 10, Siemens Healthineers, Germany) |
| **CT scan parameters** | Tube voltage | 120 kVp | 120 kVp |
|  | Tube current | 200-400 mA | 150-300 mA |
|  | Rotation time | 0.5 s | 0.5 s |
|  | Detector collimation | 128×0.625 mm or 64×0.625 mm or 32×0.6 mm | 128×0.625 mm or 64×0.625 mm or 32×0.6 mm |
|  | Arterial phase CT | 35 s after injection | 25-35 s after injection |
|  | Venous phase CT | 60 s after injection | 55-65 s after injection |
| **CT image information** | With enhanced CT | Yes | Yes |
|  | Image matrix | 512×512 | 512×512 |
|  | Field of view | 400×400 mm or 500×500 mm | 400×400 mm or 500×500 mm |
|  | Reconstruction section thickness | 1.25 mm for enhanced CT  5 mm for both enhanced and unenhanced CT | 1.25 mm for enhanced CT  5 mm for both enhanced and unenhanced CT  0.625 mm for enhanced CT |

**Table S2** **Characteristics of patients stratified by high and low risk according to** **multimodal risk score for predicting overall survival**

| **Variables** | **Training set** | | | **Validation set** | | | **Internal test set** | | | **External test set** | | |
| --- | --- | --- | --- | --- | --- | --- | --- | --- | --- | --- | --- | --- |
|  | **High risk** | **Low risk** | **P** | **High risk** | **Low risk** | **P** | **High**  **risk** | **Low**  **risk** | **P** | **High risk** | **Low risk** | **P** |
| **Number** | 45 | 313 |  | 20 | 99 |  | 21 | 98 |  | 16 | 90 |  |
| **Age in years** |  |  | 0.840 |  |  | 0.153 |  |  | 0.340 |  |  | 0.910 |
| Mean ± SD | 57.7 ± 8.1 | 57.4 ± 8.4 |  | 59.9 ± 7.4 | 57.1 ± 7.9 |  | 53.9 ± 10.0 | 56.2 ± 10.0 |  | 57.4 ± 9.3 | 57.1 ± 8.9 |  |
| **BMI (kg/m^2^)** |  |  | 0.886 |  |  | 0.143 |  |  | 0.236 |  |  | 0.095 |
| Mean ± SD | 24.0 ± 3.2 | 24.0 ± 3.0 |  | 24.6 ± 3.3 | 23.5 ± 2.9 |  | 24.1 ± 3.1 | 23.1 ± 3.4 |  | 25.7 ± 2.2 | 24.2 ± 3.5 |  |
| **NSE (ng/ml)** |  |  | 0.009 |  |  | 0.036 |  |  | 0.037 |  |  | 0.097 |
| Media (Q1 - Q3) | 15.4 (13.5 - 17.7) | 13.7 (11.9 - 15.7) |  | 14.2 (12.0 - 17.6) | 13.4 (11.7 - 14.7) |  | 14.5 (13.6 - 19.4) | 14.3 (12.2 - 15.9) |  | 14.7 (12.7 - 17.3) | 14.2 (12.3 - 16.5) |  |
| **CEA (ng/ml)** |  |  | <0.001 |  |  | 0.293 |  |  | 0.914 |  |  | 0.872 |
| Media (Q1 - Q3) | 4.5 (2.2 - 16.4) | 2.3 (1.2 - 3.7) |  | 2.7 (1.4 - 8.0) | 1.9 (1.2 - 3.3) |  | 3.3 (2.0 - 4.5) | 2.5 (1.4 - 4.0) |  | 2.0 (1.3 - 4.6) | 2.4 (1.3 - 3.9) |  |
| **MFA (cm^2^)** |  |  | <0.001 |  |  | 0.005 |  |  | 0.004 |  |  | 0.019 |
| Mean ± SD | 7.4 ± 3.9 | 5.4 ± 2.8 |  | 7.8 ± 3.9 | 5.7 ± 2.8 |  | 6.6 ± 3.1 | 5.0 ± 2.2 |  | 7.8 ± 3.0 | 5.8 ± 3.1 |  |
| **Sex** |  |  | 0.021 |  |  | 0.259 |  |  | 0.414 |  |  | 0.115 |
| Female | 18 (40.0) | 186 (59.4) |  | 9 (45.0) | 61 (61.6) |  | 10 (47.6) | 59 (60.2) |  | 4 (25.0) | 45 (55.0) |  |
| Male | 27 (60.0) | 127 (40.6) |  | 11 (55.0) | 38 (38.4) |  | 11 (52.4) | 39 (39.8) |  | 12 (75.0) | 45 (55.0) |  |
| **Smoking history** |  |  | 0.049 |  |  | 0.298 |  |  | 0.614 |  |  | 0.370 |
| Yes | 24 (53.3) | 115 (36.7) |  | 11 (55.0) | 39 (39.4) |  | 10 (47.6) | 38 (38.7) |  | 10 (62.5) | 42 (46.7) |  |
| No | 21 (46.7) | 198 (63.3) |  | 9 (45.0) | 60 (60.6) |  | 11 (52.4) | 60 (61.2) |  | 6 (37.5) | 48 (53.3) |  |
| **T stage** |  |  | <0.001 |  |  | <0.001 |  |  | 0.007 |  |  | 0.520 |
| T1 | 19 (42.2) | 266 (85.0) |  | 10 (50.0) | 83 (83.9) |  | 9 (42.9) | 77 (78.6) |  | 10 (62.5) | 66 (73.3) |  |
| T2 | 18 (40.0) | 36 (11.5) |  | 6 (30.0) | 14 (14.1) |  | 9 (42.9) | 14 (14.2) |  | 4 (25.0) | 19 (21.1) |  |
| T3 | 7 (15.6) | 9 (2.9) |  | 4 (20.0) | 0 (0.0) |  | 1 (4.7) | 4 (4.1) |  | 2 (12.5) | 5 (5.6) |  |
| T4 | 1 (2.2) | 2 (0.6) |  | 0 (0.0) | 2 (2.0) |  | 2 (9.5) | 3 (3.1) |  | 0 (0.0) | 0 (0.0) |  |
| **N stage** |  |  | <0.001 |  |  | 0.029 |  |  | <0.001 |  |  | <0.001 |
| N0 | 15 (33.3) | 268 (85.6) |  | 11 (55.0) | 80 (80.9) |  | 6 (28.6) | 76 (77.6) |  | 3 (18.7) | 73 (81.1) |  |
| N1 | 8 (17.8) | 27 (8.6) |  | 2 (10.0) | 7 (7.0) |  | 5 (23.8) | 10 (10.2) |  | 3 (18.7) | 8 (8.9) |  |
| N2 | 22 (48.9) | 18 (5.8) |  | 7 (35.0) | 12 (12.1) |  | 10 (47.6) | 12 (12.2) |  | 10 (62.6) | 9 (10.0) |  |
| **Pathological type** |  |  | 0.005 |  |  | 0.410 |  |  | 0.105 |  |  | 0.131 |
| AD | 33 (73.3) | 280 (89.5) |  | 13 (65.0) | 76 (76.8) |  | 14 (66.7) | 83 (84.7) |  | 9 (56.3) | 70 (77.8) |  |
| Others | 12 (26.7) | 33 (10.5) |  | 7 (35.0) | 23 (23.2) |  | 7 (33.3) | 15 (15.3) |  | 7 (43.7) | 20 (22.2) |  |
| **EGFR** |  |  | 0.064 |  |  | 0.457 |  |  | 0.730 |  |  | 0.433 |
| Positive | 18 (40.0) | 80 (25.6) |  | 4 (20.0) | 31 (31.3) |  | 4 (19.0) | 18 (18.4) |  | 6 (37.5) | 22 (24.4) |  |
| Negative | 27 (60.0) | 233 (74.4) |  | 16 (80.0) | 68 (68.7) |  | 17 (81.0) | 80 (81.6) |  | 10 (62.3) | 68 (75.6) |  |
| **Chemotherapy** |  |  | 0.975 |  |  | 0.601 |  |  | 0.988 |  |  | 0.717 |
| Yes | 10 (22.2) | 69 (22.0) |  | 7 (35.0) | 26 (26.3) |  | 6 (28.6) | 31 (31.6) |  | 5 (31.3) | 21 (23.3) |  |
| No | 35 (77.8) | 244 (78.0) |  | 13 (65.0) | 73 (73.7) |  | 15 (71.4) | 67 (68.4) |  | 11 (68.7) | 69 (76.7) |  |

BMI, body mass index; MFA, mediastinal fat area; NSE, neuron specific enolase; CEA, carcinoma embryonic antigen; AD, adenocarcinoma; EGFR, epidermal growth factor receptor.

**Table S3 Characteristics of patients stratified by high and low risk according to multimodal risk score for predicting** **disease-free survival**

| **Variables** | **Training set** | | | **Validation set** | | | **Internal test set** | | | **External test set** | | |
| --- | --- | --- | --- | --- | --- | --- | --- | --- | --- | --- | --- | --- |
|  | **High risk** | **Low risk** | **P** | **High risk** | **Low risk** | **P** | **High**  **risk** | **Low risk** | **P** | **High risk** | **Low risk** | **P** |
| **Number** | 52 | 306 |  | 26 | 93 |  | 28 | 91 |  | 18 | 88 |  |
| **Age in years** |  |  | 0.853 |  |  | 0.210 |  |  | 0.373 |  |  | 0.850 |
| Mean ± SD | 57.2 ± 8.0 | 57.5 ± 8.5 |  | 59.3 ± 7.5 | 57.1 ± 7.9 |  | 54.3 ± 10.7 | 56.2 ± 9.7 |  | 56.8 ± 9.0 | 57.2 ± 9.0 |  |
| **BMI (kg/m^2^)** |  |  | 0.806 |  |  | 0.284 |  |  | 0.751 |  |  | 0.098 |
| Mean ± SD | 24.1 ± 3.2 | 24.0 ± 3.0 |  | 24.3 ± 3.1 | 23.6 ± 2.9 |  | 23.4 ± 3.3 | 23.2 ± 3.4 |  | 25.6 ± 2.6 | 24.1 ± 3.4 |  |
| **NSE (ng/ml)** |  |  | 0.008 |  |  | 0.001 |  |  | 0.019 |  |  | 0.012 |
| Media (Q1 - Q3) | 15.1 (13.2 -17.8) | 13.7 (12.0 -15.7) |  | 14.7 (12.3 -19.7) | 13.0(11.7-14.7) |  | 14.9 (13.7 - 19.2) | 14.1 (12.2 - 15.6) |  | 15.0 (13.5 - 19.3) | 14.1 (12.3 - 16.4) |  |
| **CEA (ng/ml)** |  |  | <0.001 |  |  | 0.123 |  |  | 0.617 |  |  | 0.834 |
| Media (Q1 - Q3) | 4.0 (2.1 -14.9) | 2.3 (1.4 -3.7) |  | 2.9 (1.4 -8.2) | 1.8 (1.2 - 3.2) |  | 2.9 (1.8 - 4.2) | 2.3 (1.4 - 3.9) |  | 2.6 (1.2 - 5.6) | 2.4 (1.3 - 3.9) |  |
| **MFA (cm^2^)** |  |  | <0.001 |  |  | 0.011 |  |  | 0.010 |  |  | 0.018 |
| Mean ± SD | 7.3 ± 3.7 | 5.4 ± 2.8 |  | 7.4 ± 3.7 | 5.63 ± 2.9 |  | 6.3 ± 2.8 | 4.9 ± 2.3 |  | 7.7 ± 2.8 | 5.8 ± 3.1 |  |
| **Sex** |  |  | 0.031 |  |  | 0.087 |  |  | 0.447 |  |  | 0.047 |
| Female | 22 (42.3) | 182 (59.5) |  | 11 (42.3) | 59 (63.4) |  | 14 (50.0) | 55 (60.4) |  | 4 (22.2) | 45 (51.1) |  |
| Male | 30 (57.7) | 124 (40.5) |  | 15 (57.7) | 34 (36.6) |  | 14 (50.0) | 36 (39.6) |  | 14 (77.8) | 43 (48.9) |  |
| **Smoking history** |  |  | 0.052 |  |  | 0.479 |  |  | 0.595 |  |  | 0.388 |
| Yes | 27 (51.9) | 112 (36.6) |  | 13 (50.0) | 37 (39.8) |  | 13 (46.4) | 35 (38.5) |  | 11 (61.1) | 41 (46.6) |  |
| No | 25 (48.1) | 194 (63.4) |  | 13 (50.0) | 56 (60.2) |  | 15 (53.6) | 56 (61.5) |  | 7 (38.9) | 47 (53.4) |  |
| **T stage** |  |  | <0.001 |  |  | <0.001 |  |  | <0.001 |  |  | 0.106 |
| T1 | 21 (40.4) | 264 (86.3) |  | 10 (38.5) | 83 (89.3) |  | 12 (42.9) | 74 (81.3) |  | 10 (55.6) | 66 (75.0) |  |
| T2 | 21 (40.4) | 33 (10.8) |  | 10 (38.5) | 10 (10.7) |  | 9 (32.1) | 14 (15.4) |  | 5 (27.7) | 18 (20.5) |  |
| T3 | 8 (15.4) | 8 (2.6) |  | 4 (15.4) | 0 (0.0) |  | 4 (14.3) | 1 (1.1) |  | 3 (16.7) | 4 (4.5) |  |
| T4 | 2 (3.8) | 1 (0.3) |  | 2 (7.6) | 0 (0.0) |  | 3 (10.7) | 2 (2.2) |  | 0 (0.0) | 0 (0.0) |  |
| **N stage** |  |  | <0.001 |  |  | 0.012 |  |  | <0.001 |  |  | <0.001 |
| N0 | 17 (32.7) | 266 (87.0) |  | 15 (57.7) | 76 (81.7) |  | 11 (39.3) | 71 (78.0) |  | 4 (22.2) | 72 (81.8) |  |
| N1 | 11 (21.2) | 24 (7.8) |  | 2 (7.7) | 7 (7.5) |  | 5 (17.9) | 10 (11.0) |  | 3 (16.7) | 8 (9.1) |  |
| N2 | 24 (46.1) | 16 (5.2) |  | 9 (34.6) | 10 (10.8) |  | 12 (42.8) | 10 (11.0) |  | 11 (61.1) | 8 (9.1) |  |
| **Pathological type** |  |  | 0.002 |  |  | 0.320 |  |  | 0.016 |  |  | 0.083 |
| AD | 38 (73.1) | 275 (89.9) |  | 17 (65.4) | 72 (77.4) |  | 18 (64.3) | 79 (86.8) |  | 10 (55.6) | 69 (78.4) |  |
| Others | 14 (26.9) | 31 (10.1) |  | 9 (34.6) | 21 (22.6) |  | 10 (35.7) | 12 (13.2) |  | 8 (44.4) | 19 (21.6) |  |
| **EGFR** |  |  | 0.450 |  |  | 0.125 |  |  | 0.985 |  |  | 0.662 |
| Positive | 12 (23.1) | 86 (28.1) |  | 4 (15.4) | 31 (33.3) |  | 5 (17.9) | 17 (18.7) |  | 6 (33.3) | 22 (25.0) |  |
| Negative | 40 (76.9) | 220 (71.9) |  | 22 (84.6) | 62 (66.7) |  | 23 (82.1) | 74 (81.3) |  | 12 (66.7) | 66 (75.0) |  |
| **Chemotherapy** |  |  | 0.840 |  |  | 0.920 |  |  | 0.573 |  |  | 0.925 |
| Yes | 12 (23.1) | 67 (21.9) |  | 7 (26.9) | 26 (28.0) |  | 7 (25.0) | 30 (33.0) |  | 5 (27.8) | 21 (23.9) |  |
| No | 40 (76.9) | 239 (78.1) |  | 19 (73.1) | 67 (72.0) |  | 21 (75.0) | 61 (67.0) |  | 13 (72.2) | 67 (76.1) |  |

BMI, body mass index; MFA, mediastinal fat area; NSE, neuron specific enolase; CEA, carcinoma embryonic antigen; AD, adenocarcinoma; EGFR, epidermal growth factor receptor.

**Table S4** **Deep learning model** **prediction performance**

| **Variables** |  | **AUC** | **ACC (%)** | **SENS (%)** | **SPEC (%)** |
| --- | --- | --- | --- | --- | --- |
| 5-year OS | T | 0.967[0.945, 0.989] | 93.8[91.3, 96.1] | 81.9[70.2, 91.8] | 95.8[93.5, 98.0] |
|  | V | 0.911[0.880, 0.976] | 86.4[79.8, 92.4] | 66.3[44.4, 87.0] | 90.7[84.6, 96.0] |
|  | I-T | 0.819[0.693, 0.945] | 81.3[74.0, 88.2] | 62.9[40.0, 84.2] | 84.8[77.5, 91.2] |
|  | E-T | 0.775[0.660, 0.890] | 85.0[78.3, 90.6] | 56.1[30.0, 79.0] | 90.1[83.5, 95.6] |
| 3-year OS | T | 0.938[0.900, 0.976] | 88.9[85.5, 92.2] | 79.6[61.1, 94.5] | 89.5[86.0, 92.7] |
|  | V | 0.869[0.801, 0.957] | 83.3[76.5, 89.9] | 61.2[33.3, 86.7] | 86.1[79.0, 92.5] |
|  | I-T | 0.884[0.789, 0.979] | 82.3[75.6, 88.2] | 87.8[63.4, 96.4] | 81.9[74.5, 88.4] |
|  | E-T | 0.630[0.511, 0.749] | 82.1[74.6, 88.7] | 82.1[67.3, 92.4] | 84.2[77.2, 90.3] |
| 5-year DFS | T | 0.938[0.900, 0.976] | 91.6[88.5, 94.4] | 81.1[71.4, 90.5] | 94.3[91.3, 96.9] |
|  | V | 0.917[0.857, 0.977] | 89.0[83.2, 94.1] | 82.8[69.0, 96.4] | 91.0[85.1, 96.5] |
|  | I-T | 0.814[0.715, 0.913] | 75.7[68.1, 84.0] | 63.9[46.9, 80.0] | 80.3[71.6, 88.2] |
|  | E-T | 0.730[0.582, 0.878] | 71.7[62.3, 80.2] | 52.0[33.3, 72.2] | 77.8[68.3, 87.0] |
| 3-year DFS | T | 0.944[0.908, 0.980] | 91.6[88.8, 94.4] | 82.8[73.9, 91.3] | 93.7[90.7, 96.3] |
|  | V | 0.917[0.857, 0.977] | 85.7[79.8, 91.6] | 79.9[63.6, 94.1] | 87.2[80.4, 93.5] |
|  | I-T | 0.806[0.696, 0.916] | 73.9[65.5, 82.4] | 62.2[43.3, 79.2] | 77.6[68.2, 85.9] |
|  | E-T | 0.710[0.561, 0.859] | 69.8[60.4, 78.3] | 47.6[26.0, 70.6] | 75.3[65.9, 84.7] |

95% confidence intervals included in brackets. AUC, area under the ROC curve; ACC, accuracy; SENS, sensitivity; SPEC, specificity; T, training set; V, validation set; I-T, independent test set; E-T, external test set.

**Table S5 Multimodal model prediction performance**

| **Methods** |  | **AUC** | **ACC (%)** | **SENS (%)** | **SPEC (%)** |
| --- | --- | --- | --- | --- | --- |
| 5-year OS | T | 0.972[0.951, 0.993] | 91.5[88.5, 94.1] | 92.2[83.6, 98.4] | 91.4[88.3, 94.5] |
|  | V | 0.937[0.883, 0.991] | 85.6[78.9, 91.5] | 85.5[69.5, 98.7] | 85.6[77.8, 92.0] |
|  | I-T | 0.840[0.717, 0.963] | 80.6[73.1, 87.4] | 73.7[52.1, 92.9] | 81.9[74.5, 89.1] |
|  | E-T | 0.808[0.670, 0.946] | 76.4[68.8, 83.9] | 62.4[35.7, 85.7] | 78.9[70.2, 86.8] |
| 3-year OS | T | 0.950[0.915, 0.985] | 85.3[81.5, 88.8] | 91.4[87.8, 98.9] | 84.9[81.1, 88.6] |
|  | V | 0.898[0.819, 0.977] | 80.6[73.1, 87.4] | 84.9[71.5, 97.8] | 80.1[71.9, 87.1] |
|  | I-T | 0.864[0.775, 0.953] | 78.2[70.5, 85.7] | 75.8[58.5,94.2] | 77.6[69.6, 85.1] |
|  | E-T | 0.726[0.584, 0.868] | 71.6[63.2, 80.2] | 58.7[35.6, 90.3] | 73.2[64.6, 81.8] |
| 5-year DFS | T | 0.944[0.908, 0.980] | 90.7[87.7, 93.6] | 83.7[75.7, 93.6] | 92.5[89.5, 95.4] |
|  | V | 0.951[0.913, 0.989] | 89.1[83.2, 94.1] | 89.6[76.9, 98.2] | 89.0[82.6, 94.7] |
|  | I-T | 0.838[0.742, 0.934] | 78.0[70.5, 85.7] | 66.6[50.0, 82.3] | 82.4[74.4, 90.6] |
|  | E-T | 0.761[0.627, 0.895] | 70.8[622, 79.2] | 60.4[42.1, 78.2] | 74.1[64.6, 83.1] |
| 3-year DFS | T | 0.950[0.916, 0.984] | 91.0[87.9, 93.5] | 81.2[71.0, 90.0] | 93.3[90.2, 96.1] |
|  | V | 0.928[0.869, 0.987] | 85.6[79.8, 91.5] | 83.8[69.2, 95.8] | 86.1[79.3, 92.4] |
|  | I-T | 0.825[0.724, 0.926] | 77.1[68.9, 84.8] | 65.1[47.6, 82.7] | 81.0[72.5, 88.9] |
|  | E-T | 0.731[0.583, 0.879] | 69.6[61.3, 78.3] | 51.9[29.3, 72.7] | 74.0[64.2, 82.7] |

95% confidence intervals included in brackets. AUC, area under the ROC curve; ACC, accuracy; SENS, sensitivity; SPEC, specificity; T, training set; V, validation set; I-T, independent test set; E-T, external test set.
