# Supplementary material for: Multimodal Deep Learning Integrating Tumor Radiomics and Mediastinal Adiposity Improves Survival Prediction in Non‐Small Cell Lung Cancer: A Prognostic Modeling Study
Source: Cancer Med. 2025 Aug 4;14(15):e71077. doi: 10.1002/cam4.71077 (PMC12319420; doi:10.1002/cam4.71077)
Supplement: Supplementary file 4 — Appendix S2. Supplementary Methods for Image Analysis and Modeling. [file CAM4-14-e71077-s004.docx]

**Appendix Method 1. Image acquisition parameters**

Enrolled patients in two centers underwent similar scan setups but with different systems and parameters. For contrast-enhanced CT scans, patients underwent CT examinations in the axial plane after the injection of a contrast agent with a pump injector into the antecubital vein. The CT scans, covering the entire lung region, were acquired during a breath-hold with the patient supine. All the CT images were in DICOM format.

CT scans were independently reviewed by two chest radiologists with 15 years of experience who were blinded to the pathologic diagnosis and medical history of the patients. To ensure accurate capture of the nodule region, a rectangular volume of interest (VOI) containing the nodules is cropped from the CT images based on their lung nodule coordinates before training the Convolutional Neural Network (CNN). The study has obtained original CT images and localized the coordinates of lung nodules in the images. The localization results are usually represented by the coordinates of the nodule's center. Subsequently, a cube is cropped from the original CT images, with the nodule center as its central point.

**Appendix Method 2. Details of MFA measurement**

To quantify inter-reader variability, two independent radiologists (Reader A: 10 years of thoracic imaging experience; Reader B: 8 years) manually segmented MFA in a randomly selected subset of 50 cases using the semi-automatic ImageJ protocol. Intraclass correlation coefficients (ICC) were calculated for MFA. Excellent agreement was observed for MFA (ICC = 0.90, 95% CI: 0.85-0.94), confirming protocol reproducibility. Segmentation discrepancies (mean Dice score = 0.85 ± 0.07) primarily arose in regions of mediastinal fat interspersed with vascular structures, which were resolved by consensus review.

**Appendix Method 3. DL model training process**

To evaluate the performance of CNNs across datasets with different distributions, we tested several classic CNN architectures, including ResNet-50, ResNet-101, Inception-v3, VGG-16, DenseNet-121, and DenseNet-169. The batch size was set to 32, with a learning rate of 1e-6, and a dropout rate of 0.2 was applied to prevent overfitting. Early stopping was used, with a patience of 10 epochs based on the validation set loss. The network was trained for 100 epochs.

To mitigate the risk of overfitting during training, we implemented various techniques, including data augmentation, early stopping, and learning rate decay. The data augmentation process involved random rotations around the lesion center (from -180° to 180°), random shifts of the lesion by 3 pixels, random sharpening, and random blurring.

**Appendix Method 4. Multimodal model training process**

The multimodal model was built on Cox regression, a widely utilized model for survival prediction. It was first trained using the training set and evaluated on the validation set, followed by deployment on both internal and external test sets. The model inputs consisted of pretreatment MFA and the risk scores calculated by the DL model, both treated as continuous variables. Two separate Cox regression models were trained to predict OS and DFS, with an L2 regularization penalty set to 0.1.
